# Making Sense of Complex Carbon and Metal/Carbon Systems by Secondary Electron Hyperspectral Imaging

**DOI:** 10.1002/advs.201900719

**Published:** 2019-08-07

**Authors:** Kerry J. Abrams, Maurizio Dapor, Nicola Stehling, Martina Azzolini, Stephan J. Kyle, Jan Schäfer, Antje Quade, Filip Mika, Stanislav Kratky, Zuzana Pokorna, Ivo Konvalina, Danielle Mehta, Kate Black, Cornelia Rodenburg

**Affiliations:** ^1^ Department of Materials Science and Engineering Sir Robert Hadfield Building The University of Sheffield Mappin Street Sheffield S1 3JD UK; ^2^ European Centre for Theoretical Studies in Nuclear Physics and Related Areas (ECT*‐FBK) Trento 38123 Italy; ^3^ Trento Institute for Fundamental Physics and Applications (TIFPA‐INFN) Povo Trento 38123 Italy; ^4^ Leibniz Institute for Plasma Science and Technology (INP Greifswald e.V.) Felix‐Hausdorff‐Str. 2 17489 Greifswald Germany; ^5^ Institute of Scientific Instruments of the CAS Královopolská 147 612 64 Brno Czech Republic; ^6^ School of Engineering University of Liverpool Harrison Hughes Building Liverpool L69 3GH UK

**Keywords:** carbon orientations, carbon surface analysis, characterization, modeling, secondary electron emission, secondary electron hyperspectral imaging, secondary electron spectroscopy

## Abstract

Carbon and carbon/metal systems with a multitude of functionalities are ubiquitous in new technologies but understanding on the nanoscale remains elusive due to their affinity for interaction with their environment and limitations in available characterization techniques. This paper introduces a spectroscopic technique and demonstrates its capacity to reveal chemical variations of carbon. The effectiveness of this approach is validated experimentally through spatially averaging spectroscopic techniques and using Monte Carlo modeling. Characteristic spectra shapes and peak positions for varying contributions of sp^2^‐like or sp^3^‐like bond types and amorphous hydrogenated carbon are reported under circumstances which might be observed on highly oriented pyrolytic graphite (HOPG) surfaces as a result of air or electron beam exposure. The spectral features identified above are then used to identify the different forms of carbon present within the metallic films deposited from reactive organometallic inks. While spectra for metals is obtained in dedicated surface science instrumentation, the complex relations between carbon and metal species is only revealed by secondary electron (SE) spectroscopy and SE hyperspectral imaging obtained in a state‐of‐the‐art scanning electron microscope (SEM). This work reveals the inhomogeneous incorporation of carbon on the nanoscale but also uncovers a link between local orientation of metallic components and carbon form.

## 1 Introduction

Films and nanomaterials made of complex carbon systems are key to many next‐generation technologies^1^, ^2^, ^3^ and scientists are keen to exploit their functionalities, for example in batteries and electrode surfaces.^4^, ^5^, ^6^ Their versatility originates from the strong dependence of their physical properties on the ratio of sp^2^‐graphite like bonds to sp^3^‐diamond‐like bonds.^7^ There are many forms of sp^2^‐bonded carbons with various degrees of graphitic ordering, ranging from microcrystalline graphite to glassy carbon. During the fabrication of carbon film based devices, electron irradiation is prevalently used for nanopatterning, nanostructure characterization and surface modification.^8^ Compositional changes on the nanoscale on carbon surfaces are often difficult to elucidate, as available techniques can modify the surface of interest. However, knowledge of these surfaces and their interaction with the environment are imperative for applications such as battery materials,^9^ electrode materials, electrocatalysis and imaging of biomolecules.^10^ Typically, carbon materials are characterized by techniques such as Raman, X‐ray photoelectron (XPS), ultraviolet photoelectron spectroscopy (UPS), and Fourier‐transform infra‐red (FTIR) spectroscopy but these techniques all have inherent limitations associated with spatially averaging techniques.^11^, ^12^, ^13^, ^14^ There is still limited understanding of the chemistry of the contaminants, especially at the early stages of contamination at sub‐monolayer contamination thickness, for which UPS is most sensitive.^13^ That such sub‐monolayer contamination can lead to significant changes in the secondary electron (SE) emission spectra was shown on silicon.^15^

Here, we probe carbon surfaces with a novel spectroscopy technique based on the energy‐selective collection of secondary electrons which has been used for insights into nanoscale mapping of semicrystalline polypropylene,^16^ organic photovoltaics,^14^, ^17^ hybrid solar cells,^18^ and hierarchical biopolymers.^19^, ^20^ SE spectroscopy was found to be an effective tool for carbon material characterization as long ago as 1970s.^21^, ^22^ Our equipment is based in a low voltage scanning electron microscope (LV‐SEM) and images can be formed using SEs with specified energy ranges. As the energy range can be adjusted by the user, stacks of images each with a different energy range can be collected and the SE spectra derived, we refer to this technique as Secondary electron hyperspectral imaging (SEHI). SEHI allows imaging at a higher resolution, i.e., via the removal of topography^23^ or from the distinctive peaks in the SE spectrum isolating elemental information.^24^ Therefore, surface information can be produced as a function of electron energy and information below the nanoscale is obtained without the need for electron‐transparent lamella. Highly oriented pyrolytic graphite (HOPG) is expected to be pure sp^2^ and often used as calibration material for scanning tunnel microscopy due to having large, flat, chemically inert area.^25^ Graphite materials are sensitive to unintentional contamination from hydrocarbons in ambient air^26^ and during analysis in the SEM, the evolution of this carbonaceous species by electron beam ion deposition (EBID) resulting in significant reductions in image resolution which can block the original surface from view affecting investigations of the surface.^27^ Further, probing techniques used to characterize carbon surfaces often utilize high beam currents which can also induce EBID, e.g., nano‐Auger spectroscopy typical uses currents of 10^−6^ Ampere^28^ whereas SEHI uses currents which are in the picoAmpere range. Thus, with these small primary beam currents and short exposure times, the onset of EBID contamination is delayed and so native carbon surfaces revealed.

The aim of this paper is to probe the character of different carbon species on HOPG surfaces by SEHI to obtain further understanding of the inherent bond types and their associated SE emission. These insights are then applied to carbon present within printed complex metal carbon systems which are expected to affect the end functionality dependent on the bond type of local carbon inclusions. The experimental results are corroborated by Monte Carlo (MC) simulations.

## 2 Results and Discussion

### 2.1 Evolution of Carbon on Carbon

**Figure 1:**
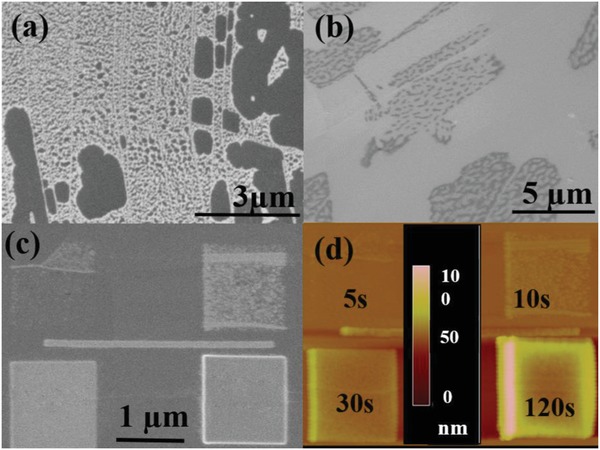
a) SEHI micrograph using all secondary electron energies up to 6 eV of fresh HOPG and b) SEHI of aged HOPG using all secondary electron energies up to 6 eV. c) SEM micrograph of HOPG surface with EBID boxes. d) Corresponding atomic force microscopy (AFM) micrograph with the height bar denoting the height of the EBID box in nm. The same scale bar is valid for (c) and (d).

HOPG, a manmade polycrystalline graphite (stacked graphene) is well characterized and, as it is possible to exfoliate the top surface revealing “clean” or “fresh” layers, is often used as calibration material for scanning tunnel microscopy.^25^ However, this work reveals localized contaminants in **Figure** **1**a,b on HOPG in line with previous reports of contamination on graphitic surfaces.^27^, ^29^

The difference between Figure 1a,b is that these SEHI (<6 eV) micrographs of HOPG were taken after different exposures to air. Figure 1a shows a newly exfoliated (fresh) surface in which the terraces and grains are decorated with bright white islands, which are dominant on the terraces or grain boundaries but are also observed within the grains. Figure 1b shows the same HOPG sample but after exposure to air for 5 days (aged) resulting in a different appearance with extended bright areas now being exhibited by many grains. The original bright islands now appear interconnected. However, some grains still exhibit a few dark areas which are surrounded by bright contrast. This white contrast is dynamic and differs from the appearance of the typical contamination seen in EBID. Figure 1c,d show typical EBID boxes formed on a HOPG surface with different electron doses of 6.1 and 12.2 C m^−2^, both produce a non‐uniform nucleation of contaminations on the surface following existing localized features on the HOPG surface. The higher doses of 36.7 and 146 C m^−2^ produced more uniform boxes of a carbonaceous species. The height of the EBID increases from 0 to ≈100 nm with dose as shown in the atomic force microscope image in Figure 1d. Therefore, the evolution of contamination on fresh HOPG surfaces at room temperature (RT) within the vacuum chamber of an SEM is observed to be a two‐stage process. Hereafter, these two stages will be referred to as primary (low dose with localized increased emission in the nanoscale bright areas) as in Figure 1a,b and Secondary contamination (manifested by the presence of a continuous contamination layer with decreased electron emission) as in Figure 1c.

**Figure 2:**
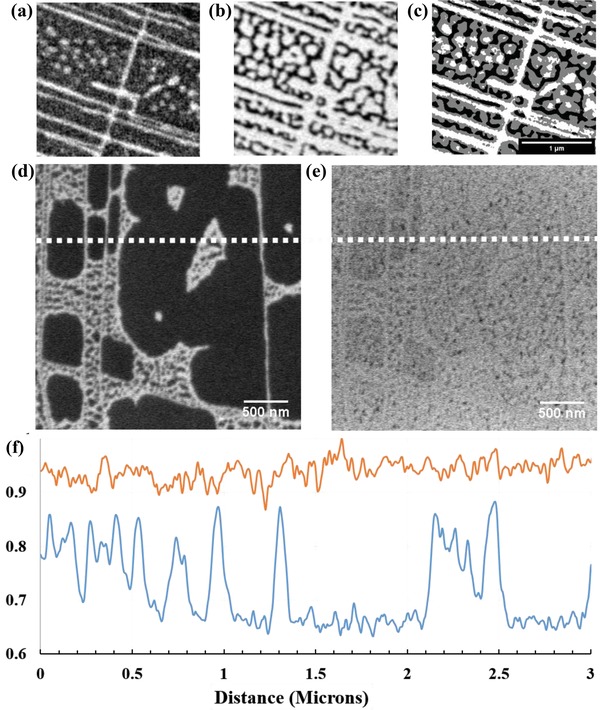
a) Micrographs of primary contamination of a fresh surface of HOPG. Image taken using all energies up to 6 eV at time = 0, b) micrographs of primary contamination of a fresh surface of HOPG. Image taken using all energies up to 6 eV at time = 4 min and dose 0.4 C m^−2^), c) overlay of the images at the start and end illustrating how the bright contrast traverses across the HOPG surface, and d) micrographs of Secondary contamination of a Fresh surface (Pre‐EBID). Image has been contrast enhanced. e) Post EBID‐same area as d) illustrating the loss of contrast observed with electron beam exposure. f) Intensity line profiles taken from the areas marked by dashed white lines of d) Pre‐EBID in blue and e) Post EBID showing the loss of emission observed with Secondary contamination in orange.

**Figure** **2** shows both stages of contamination at a higher magnification. Figure 2a,b are micrographs of the same area (taken from a times series) and show the evolution of the primary contamination, which begins with an inhomogeneous contrast across the surface (as in Figure 1a and at higher magnification in Figure 2a). The grain edges exhibit increased electron emission with some white islands (≈100 nm) observed within grains. The white contrast traverses from the initial islands across the surface and leads to a complex patterning as seen in Figure 2b. Figure 2c shows an overlay of the two images illustrating how the white contrast from the islands traverses across the HOPG surface (shown on the overlay in grey) and terminates in an inhomogeneous contrast with hexagonal and zig–zag shaped edges (Low magnification micrographs can be found in Section S3 in the Supporting Information.) Similar surface features on HOPG surfaces have been observed using electrostatic force microscopy by Lu et al.^30^ In their work they correlate the coexistence of insulating and conductive behavior on graphite surfaces to differences in electrical potentials on the surface, which is consistent with our observation of large differences SE emission intensities between the different carbon surface conditions.

The Secondary stage of contamination is observed in Figure 1c,d and Figure 2d,e where the localized bright features are no longer visible and the concomitant appearance of an additional material and a midgray contrast is exhibited. At this stage, when the magnification of the image is reduced, a darker rectangle is observed over the previously scanned region. This is the typical EBID^27^, ^31^, ^32^ reported to consist of hydrogenated amorphous carbon species which results in an reduction of intensity as observed in the line profile of Figure 2f (orange and blue plots of the green dashed lines in Figure 2d,e). It is well known that this EBID is deposited through the polymerization of any hydrocarbon molecules that are adsorbed on the surface leading to the build‐up of a carbonaceous species in the exposed area and is known to degrade specimen images in transmission and scanning electron microscopies.^33^ While EBID can be exploited to create carbon pillars^34^ or weld nanoparticles,^35^ the presence of an EBID related carbon species here prevents further probing of the primary stage of contamination. Therefore, in this work all further analysis on these carbon surfaces was kept below the exposure required for EBID to ensure that the native surfaces are probed.

**Figure 3:**
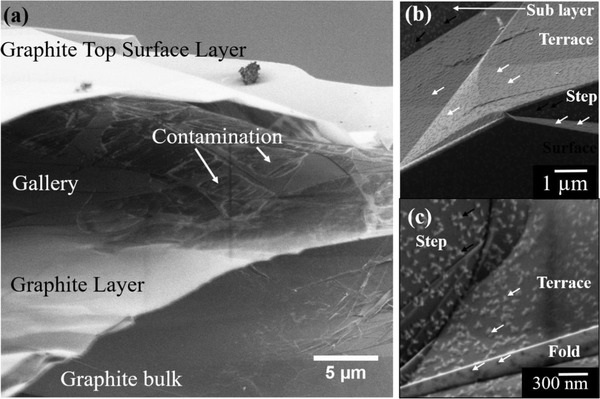
a) Low Voltage SEM (LVSEM) micrographs of HOPG taken looking at the edge of the sample where the layers have separated and ambient air contamination has intercalated into the gallery of HOPG. b) Cryo‐LV SEM micrograph of contamination islands on all surfaces and features on HOPG surface. c) High resolution Cryo‐LV SEM micrograph of contamination islands.

Our observations also revealed the presence of another type of contamination. **Figure** **3**a shows an SEM micrograph of a HOPG sample edge and shows the presence of intercalated contamination (highlighted by orange arrows) in the gallery of the graphite layers. This contamination is assumed to be due to the unavoidable ambient air exposure over time since manufacture, as such, the age of this contamination is difficult to ascertain. The typical analysis of the HOPG is carried out on the top layer at a distance from the edge but any existing contamination within the graphite gallery or on the underlayers while not visible could affect the response of the material to the electron beam. Investigations at cryoconditions are often used to reduce the contamination issue,^36^ Figure 3b,c show cryo‐LV SEM micrographs of HOPG at low and high magnification respectively. Contamination islands can still be observed across the whole of the surface on the fresh surface, they are seen on all the typical topographical features of HOPG such as steps (highlighted by orange arrows), terraces (highlighted by green arrows) and folds (highlighted by brown arrows. Note that the thin fold shows the contamination islands as black on a brighter substrate as the contrasts come from the transmission of the primary beam and is unrelated to SE emission. However, the islands appear brighter than the substrate on the steps, surfaces, and terraces as the contrast here originates from an increased SE emission. This contamination can be minimized with the exfoliation of top layers but cannot be totally removed, therefore is an artifact that could not be avoided and therefore one needs to be aware when analyzing bulk HOPG surfaces.

### 2.2 SE Spectroscopy of Carbon on Carbon

The observation of distinct grey levels for fresh HOPG (brightest emission), aged HOPG (lowest emission) and EBID (midgray level) surface indicates the sensitivity of SE emission to the configuration of the carbon which should also be reflected in differences in their SE spectral shape. The electron transport within these materials is key to their analysis^10^ and so SE spectra were collected of these three types of HOPG surfaces, i.e., fresh, aged, and EBID.

**Figure 4:**
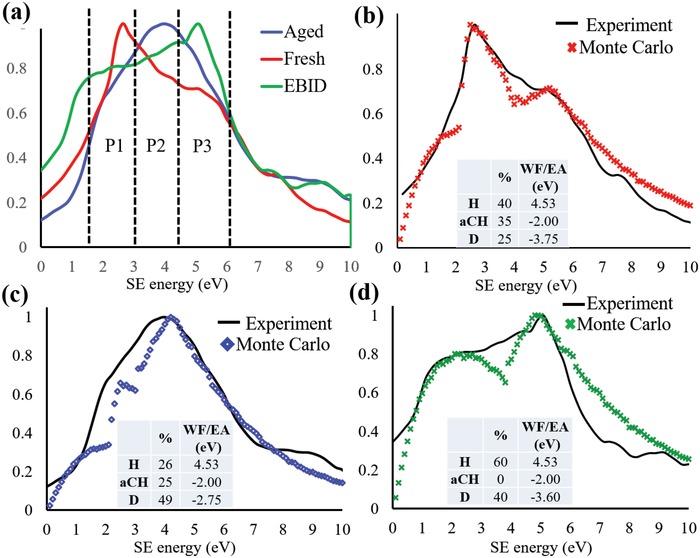
a) SE Spectrum of a fresh HOPG surface (red), an aged surface (blue) and HOPG surface with an EBID layer (green), b) Monte Carlo plot of SE spectrum of a fresh HOPG surface (red) with experimental curve (black.) Table inset shows the modeled proportion of HOPG, a‐CH, and Diamond and their work functions, c) Monte Carlo plot of SE spectrum of an aged HOPG surface (blue) with experimental curve (black) Table inset shows the modeled proportion of HOPG, a‐CH, and Diamond and their work functions; d) Monte Carlo plot of SE spectrum of an HOPG surface with EBID (green) with experimental curve (black) Table inset shows the modeled proportion of HOPG, a‐CH, and Diamond and their work functions.

The SE spectra of fresh, aged and EBID covered surfaces of HOPG reveal substantial differences as can be seen in **Figure** **4**a. The dominant peak position is observed to shift to a higher energy with various stages of contamination, i.e., fresh, aged (thin layer of primary contamination), and EBID (a thick layer of secondary contamination.) Here, the spectrum of fresh HOPG has a dominant peak (labeled P1) in the energy range 2–3.3 eV, ≈ 2.7 eV. When the HOPG surface is aged, the dominant peak shifts up to the higher energy of ≈4.2 eV (labeled P2) and if an EBID induced carbonaceous species is present, then the dominant peak moves to (labeled P3) ≈5.5 eV. The Table S2 in the Supporting Information shows the expected values from the literature for HOPG and Diamond with sp^2^ and sp^3^ bond types and confirms the SE emission is different for each allotrope with each dominant peak in a different energy range. Therefore, we assign P1 to be sp^2^‐like, P3 to be sp^3^‐like and P2 to be associated with the bond‐type associated with the nature of the primary contamination. Amorphous hydrogenated Carbon (a‐CH) is cited to have a higher SE yield than HOPG, due to its diamond‐like sp^3^ bonding and hydrogen content and their respective low work functions.^37^ This is explained by the formation of an energy band gap and surface states that help the secondary electrons to diffuse and tunnel through the surface energy barrier. We propose that the primary contamination is related to the chemisorption of hydrogen, which is known to preferentially initiate at defects and cleaved graphite surfaces.^38^ This acts to change the work function of the surface (resulting in an increased electron emission) and additionally buckles the HOPG surface which will convert the graphitic sp^2^ bonds to more distorted sp^3^‐like bonds.^39^ This assertion is in agreement with other researchers.^13^, ^29^, ^38^, ^39^ Salim et al.^13^ additionally points out that understanding the surface of HOPG surfaces are key to many applications and concluded that UPS is better than XPS. A link between UPS and SE spectra has previously been demonstrated^18^ and that by using a well‐defined energy range of SE to collect scanning electron microscopy images sub‐nanometer resolution can be obtained on polymer blends. Thus, the sensitivity of SE spectra is not surprising because adsorbed molecules can result in dipoles that can increase or reduce the surface barrier for electrons leaving the material. Hydrogen adsorption is known to lower the work function of HOPG by 0.6 eV as measured by Ruffieux et al.^40^ A reduced work function allows more SE to escape, thus areas that exhibit hydrogen adsorption appear brighter than those free of adsorption or those that contain oxygen in the form of ‐OH surface groups. The latter surface functional groups increase the work function of HOPG by up to 1.02 eV^41^ thus such functionalized areas appear dark as the number of SEs that can overcome the surface barrier is reduced. Thus, we ascribe the brightest areas in Figure 1 to result from adsorbed hydrogen.

The dominant peak in the SE spectra of aged HOPG appears at a higher energy range closer to the energy range for sp^3^ bond types already observed in dedicated surface science SE instrumentation.^42^ With the assumption that the primary and Secondary contamination is made up from three components‐ sp^2^‐like HOPG, a‐CH, and sp^3^‐like diamond, MC simulations of SE emission of HOPG surfaces were generated with different ratios of these carbon types to match the shape of the SE spectra obtained in our SEM, as in Figure 4b–d). While our system is not a perfect spectrometer system, it has the advantageous capability to image the surfaces over micrometer areas and so the ratios of areas with different emission characteristics can then be used as an input for the MC model spectra (see Section S3 in the Supporting Information.) The modelled spectra and the associated percentages of HOPG, amorphous hydrogenated Carbon and Diamond (labelled H, a‐CH, and D in the inset table) are shown in Figure 4b–d. The modeled spectra correlate with the experimental spectrum and confirm that the SE emission is sensitive to the different carbon bond type and contamination character. The match of the experimental and the simulated MC SE spectra with contamination coverages observed in low magnification SEM micrographs (Figure S3, Supporting Information) both validates and refines our peak allocation in terms of sp^2^‐like, a‐CH, and diamond sp^3^‐like. For further experimental validation, Raman and XPS of the different HOPG surfaces was performed, shown in Sections S4 and S5 in the Supporting Information. The Raman analysis reveals a change in disorder (a change in the *I*_d_/*I*_g_ ratio) however, this parameter is restricted when evaluating the amorphous nature of the investigated carbons, as it is only indirectly related to the fraction of sp^3^ sites.^6^ The XPS on fresh and aged HOPG confirmed a very small increase in disordered carbon and sp^3^ content with aging, however once again, this technique is expected to be affected by inhomogeneous depth‐resolved distributions of the different bond‐types.^43^, ^44^ From this, we conclude that SEHI reveals additional key information about sp^2^ and sp^3^ ratios and has significant potential as another tool for the characterization of carbon surfaces, especially if carbon is present within more complex systems such as those in carbon‐containing metal films.

### 2.3 Investigations of Metal/Carbon Films

**Figure 5:**
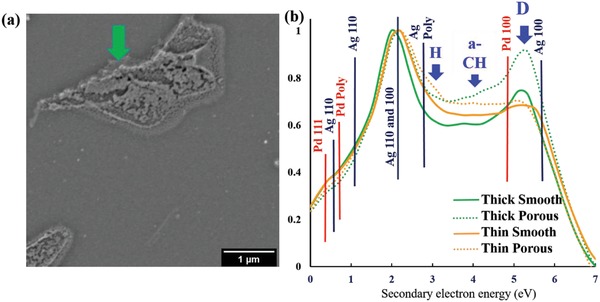
a) LVSEM of PdAg film exhibiting both smooth and porous morphologies. b) SE spectrum of thin and thick PdAg metal films.

Having linked the variations in emission from different energy regions to the presence of different carbon bonding states, the inspection of those different carbon bond types in a more complex system is now possible. We now look to a less well studied metal/carbon reactive organometallic (ROM) system. **Figure** **5**a shows an SEM micrograph of a printed PdAg film on silicon which consists of areas of a porous morphology (highlighted by green arrow) and a smoother morphology. The existence of porous and smooth areas in ROM deposited films was previously observed in an Ag ROM system, whereby the conductivity was found to be influenced by the carbon content and its microstructure.^45^ The origin for these localized morphological variations is yet unknown but can be elucidated on by the SE spectra as shown in Figure 5b which contains SE spectra collected from the smooth and porous regions of two different thickness films.

In Figure 5b, the SE peaks of the metals are labeled according to the literature (Table S2.2, Supporting Information) whereas the dominant peak values for the carbon components are taken from our MC modeling results in Figure 4 (highlighted with blue arrows). The metal films all have a dominant peak at 2 eV, literature assigns this emission energy to the Ag 110 and 100 directions.^46^, ^47^ The SE Spectra reveal significant differences in the spectral regions associated with the carbon component contributions, i.e., HOPG sp^2^ like, a‐CH mixed, and Diamond sp^3^‐like. As shown in Section S6 in the Supporting Information, energy dispersive x‐ray spectroscopy (EDX) analysis reveals limited information except the atomic percentage of carbon was higher in the porous regions compared to the smooth regions, in agreement with our SE spectra. However, the SE spectra reveal further important differences in the character of the carbon species within the metal films. In the case of the thin films, SE emissions from each carbon bond type are present but contributions from the sp^2^‐like and a‐CH mixed components are increased in the porous form. The spectra of the thicker films reveal the presence of carbon too, albeit with a different character to that of the thin film. The sp^2^ peak region in the thin film is higher than in the thick film whereas, the thick film exhibits a strong SE emission in the region ascribed to carbon in its sp^3^ form. This suggests that different crystal orientations in the metal might interact or be correlated to different bond types of carbon. The carbon bond type will contribute to or be detrimental to the conductivity of the film, for example, graphite (sp^2^‐like) is conductive whereas diamond (sp^3^‐like) is insulating. However, these higher energy regions are also likely to be influenced by the presence of Pd (100) and Ag (100) peaks, so further work would require SE spectra to be collected with a higher energy resolution to make this distinction clear. Further work would also be needed to use the above information to maximize the conductivity by reconstructing the localized nucleation and growth processes based on a more detailed SE spectroscopy study in combination with more traditional characterization techniques. Nevertheless, we have demonstrated that the SE spectroscopy in an SEM with a through‐the‐lens detector can provide localized information not accessible by traditional averaging techniques such as Raman, XPS, and EDX.

## 3 Conclusions

SEHI analysis of HOPG surfaces in LV‐SEM revealed a two stage process in surface modification of HOPG through hydrogenation (primary) and charge induced deposition of amorphous carbon species (Secondary). This insight into carbonaceous film formation on carbon has elucidated on different carbon bond types and is also reflected in the changing shape of SE spectra which are confirmed by MC modeling. Exploring SE spectral features to identify the presence of carbon contamination hidden in films deposited from ROM inks reveals links between carbon incorporation, its orientation, and the crystal orientation of the metallic component. As the link between carbon with different bond types and metal deposition is of interest to other complex systems such as anodes in Li‐ion batteries or in catalysts,^9^ the application of SEHI could hold the key to an improved understanding and subsequent optimization of material systems. SEHI also has the capability of revealing chemical changes on the nanoscale directly observable on bulk materials with the proviso that the spectra collection is carried out in a collection regime that prevents EBID.

## Conflict of Interest

The authors declare no conflict of interest.

## Supporting information

Supplementary
