# Supplementary material for: Making Sense of Complex Carbon and Metal/Carbon Systems by Secondary Electron Hyperspectral Imaging
Source: Adv Sci (Weinh). 2019 Aug 7;6(19):1900719. doi: 10.1002/advs.201900719 (PMC6774015; doi:10.1002/advs.201900719)
Supplement: Supplementary file 1 — Supplementary [file ADVS-6-1900719-s001.pdf]

Copyright WILEY-VCH Verlag GmbH & Co. KGaA, 69469 Weinheim, Germany, 2019.

ADVANCED SCIENCE
Open Access

Supporting Information

for *Adv. Sci.,* DOI: 10.1002/advs.201900719

Making Sense of Complex Carbon and Metal/Carbon Systems by Secondary Electron Hyperspectral Imaging

Kerry J. Abrams, Maurizio Dapor, Nicola Stehling, Martina Azzolini, Stephan J. Kyle, Jan S. Schäfer, Antje Quade, Filip Mika, Stanislav Kratky, Zuzana Pokorna, Ivo Konvalina, Danielle Mehta, Kate Black, and Cornelia Rodenburg*

Supplementary Information

Making Sense of Complex Carbon and Metal/Carbon systems by Secondary Electron Hyperspectral Imaging

Kerry J Abrams,[1] Maurizio Dapor,[2] Nicola Stehling, [1] Martina Azzolini, [2] Stephan J Kyle, [1] Jan S Schäfer,[3] Antje Quade, [3] Filip Mika[4], Stanislav Kratky[4], Zuzana Pokorna[4], Ivo Konvalina[4], Danielle Mehta,[5] Kate Black,[5] and Cornelia Rodenburg[1]

1 Department of Materials Science and Engineering, Sir Robert Hadfield Building, Mappin Street University of Sheffield, UK.

2 European Centre for Theoretical Studies in Nuclear Physics and Related Areas (ECT*-FBK) and Trento Institute for Fundamental Physics and Applications (TIFPA-INFN) Trento 38123, Italy

3 Leibniz Institute for Plasma Science and Technology (INP Greifswald e.V.), Felix-Hausdorff-Str. 2, 17489 Greifswald, Germany

4 Institute of Scientific Instruments of the CAS, Královopolská 147, Brno, Czech Republic

5 School of Engineering, University of Liverpool, Harrison Hughes Building, Liverpool, L69 3GH

Experimental

Sample preparation -Highly oriented pyrolytic graphite (HOPG) sample (Agar Scientific-Mosaic ±3.5%) was observed as a) Fresh b) Aged. Fresh surfaces are defined as the newly revealed layer post mechanical exfoliation and were inserted into the microscope sample chamber within 1 minute. Aged surfaces are native surfaces left within air for a prolonged time

before observation. Complex metal carbon films on silicon were PdAg and produced by ROM were supplied by University of Liverpool.

SEHI- Generation of signature SE spectra-A Helios Nanolab G3 UC microscope specifically designed for ultrahigh resolution at low voltages (<1KV) was used to observe the carbon allotropes. Unlike ordinary SEM analysis, no conductive coating was deposited onto the samples prior to probing carbon surfaces, providing real surface information. An accelerating voltage of 1 kV, typical vacuum pressure =10^{-6} mbar, current = 50 pA and a working distance of 4 mm was used. The design of the in-lens detector of this SEM allows the collection of different secondary electron (SE) energy ranges by changing a mirror electrode voltage (MV) with a tube bias of 150V.[1] Schematic of this procedure can be found in figure SI 1. Routine plasma cleaning of the SEM chamber was performed to reduce unwanted contamination of carbon surfaces.[2]

Hyperspectral Imaging -An automatic iFast collection recipe [3] was utilized to step the MV between -15V and 15 V (energy range of -0.7 to 12.7 eV) and an image (frame time 0.5 seconds) was collected with each successive 0.5 V (this corresponds to an electron energy step size of ~0.2 eV). The energy calibration is discussed further in Supplementary information SI1.

Spectrum Output-Each region of interest was processed through an in-house MATLAB procedure which outputs the mean and differentiated intensity levels for each MV step.

Peak Analysis- Multiple spectra of each HOPG surface were collected to ensure the spectra representative and reproducible. The 3 specific peaks were labelled P1, P2 and P 3. Comparison to literature shows that the SE emission of different carbon allotropes exhibit dominant peaks in

different energy ranges below 10 eV. Table SI 2.1 shows that HOPG which is expected to be sp^2 has a dominant peak around 3eV and diamond with sp^3 bond type exhibits a peak around 6 eV.

Monte Carlo Modeling-The Monte Carlo code is based on calculations of the differential elastic scattering cross section performed by the Relativistic Partial Wave Expansion Method (RPWEM). This method consists of looking for the numerical solution of the Dirac equation in a central field and using it to calculate the phase shifts of the scattered wave and the scattering amplitudes. The values of the total elastic scattering cross section of 1000 eV electrons in C calculated using the RPWEM is 0.535 10^{-16} cm^2. The first transport cross section is 0.0390 10^{-16} cm^2 and the second transport cross section is 0.0814 10^{-16} cm^2. The energy straggling and the inelastic scattering cross sections are calculated by using the dielectric function model. The inelastic mean free paths for 1000 eV electrons impinging on amorphous carbon is 23.5 10^{-8} cm, on diamond 17.0 10^{-8} cm, and on graphite 19.5 10^{-8} cm. The backscattering coefficient of a 1000 eV electron beam impinging on a C target is approximately 0.14. Note that, while diamond and amorphous carbon are isotropic crystals, HOPG is an anisotropic crystal. In particular, it is a uniaxial crystal with layered structure. For such uniaxial anisotropic crystals, the dielectric function ε is a tensor with only two different diagonal elements, one perpendicular ($\varepsilon_{perpendicular}$) and the other parallel ($\varepsilon_{parallel}$) to the c -axis. The former is associated with the response for a momentum-transfer perpendicular to the c -axis (in-plane excitations), the latter with the response for a momentum transfer parallel to the c -axis (out-of-plane excitations). To describe the HOPG inelastic scattering, we assume that a single inelastic collision is ruled either by the differential inelastic scattering cross section corresponding to $\varepsilon_{parallel}$ or by the one corresponding to $\varepsilon_{perpendicular}$. We then introduce a numerical parameter, *f*, ranging from 0 to 1. During the simulation of electron transport in HOPG, for each inelastic collision, a random number

uniformly distributed in the range (0, 1) is generated. If the random number is lower than the value of *f*, the collision is ruled by the differential inelastic scattering cross section corresponding to $\varepsilon_{parallel}$, otherwise it is ruled by the differential inelastic scattering cross section corresponding to $\varepsilon_{perpendicular}$. According to Ref[4] , we used, for the present simulations, f = 0.4. The simulated spectra are compared with the experimental data taking into account the simultaneous presence of HOPG, a-CH and diamond on the sample surfaces. So, depending on the sample history, we have combined the spectra corresponding to the three components assuming different values of the percentages of the three materials. Also the negative electron affinities of diamond have been selected in order to take into account of the differences in the H contamination of diamond due to the sample history. The values of the percentages and of the WF/NEA of the fresh, aged, and EBID samples are summarized in the inset tables of figure 4 in the main document. Note that the slope breakdown in the Monte Carlo plot is a consequence of the NEA of amorphous carbon and of diamond. In fact, the NEA of amorphous carbon is -2eV, while the NEA of diamond ranges, in our samples, from -2.75eV to -3.75eV.

Raman Spectroscopy Analysis-Raman spectroscopy was conducted using an inVia Raman microscope (Renishaw, United Kingdom) using a 514 nm laser set to 20mW. The peaks were plotted from 0-4200 cm-1 and peak fitting was completed in the regions expected to reveal disorder in HOPG.

XPS Analysis-The elemental surface composition and chemical binding properties were analyzed by X-ray photoelectron spectroscopy (XPS) using an AXIS Ultra DLD electron spectrometer (Kratos Analytical, Manchester, UK). The spectra were recorded utilising monochromatic X-rays Al kα (1486.6 eV) with a medium magnification lens mode and by selecting the slot mode,

providing an analysis area of approximately 250 µm in diameter. A pass energy of 80 eV was used for estimating the chemical elemental composition and 10 eV for the highly resolved measured C 1s peaks to investigate chemical functional groups. Charge neutralization was used for all samples to reduce any potential differential charging effects. Data acquisition and processing were carried out using CasaXPS software, version 2.15 (Casa Software Ltd., UK).

Energy Dispersive Xray analysis- Samples were put in Nova nanosem 400 with Oxford Instruments (Insert) without conductive coating and probed with 5kV to excite the K lines of the Pd, Ag C and O.

SI 1) SEHI technique and Energy calibration

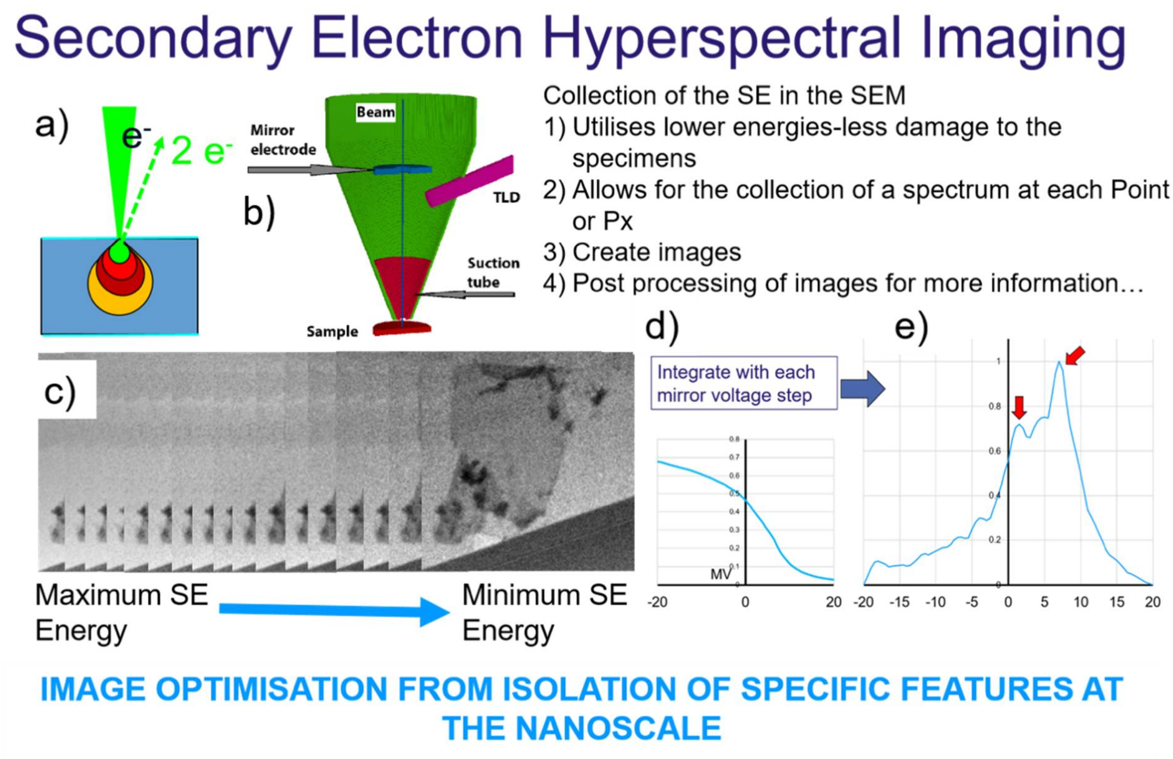

Figure SI 1 Schematic of the SEHI technique a) Secondary electrons are produced when a primary beam hits the surface b) The set-up of the through the lens system that allows energy selection of electrons c) Hyperspectral array of intensities d) S Curve obtained by plotting the intensities from maximum to minimum energy e) The integration of the S Curve with respect to each voltage step produces a signature SE spectrum.

SE energy calibration for this system can be found in the supporting information of reference Wan *et al.* [5] Graphene is single layer of carbon atoms in 2D space and ordered along the basal plane and in a multilayer graphene sample, the dominant peak in the SE spectrum is observed to shift to a higher energy[6]

SI2 Secondary electron energy emissions of Carbon allotropes and Metals Pd and Ag.

Material	Dominant Peak (eV)	Literature
**HOPG (Sp^2 Dominant)**	3	Ueno, Willis, Ferron
**Glassy Carbon (Amorphous)**	4	Ueno
**Diamond (Sp^3 Dominant)**	6	Hoffman

Table SI 2.1 Expected SE peaks from different carbon materials with different dominant bond types. [7–10]

Material	Dominant Peak (eV)	Literature

**Pd Polycrystalline**	0.88	Lang
**Pd 111**	0.5	Lang
**Pd 100**	4.7	Lang
**Ag Polycrystalline**	2.7	Otto
**Ag 100**	2.0, 5.8	Novolodskii, Otto
**Ag 110**	0.6, 1.2, 2.0	Novolodskii, Otto

Table SI2.2 Expected SE peaks from Palladium and Silver from various references.[11–13]

SI3 Contamination of HOPG surface analysis

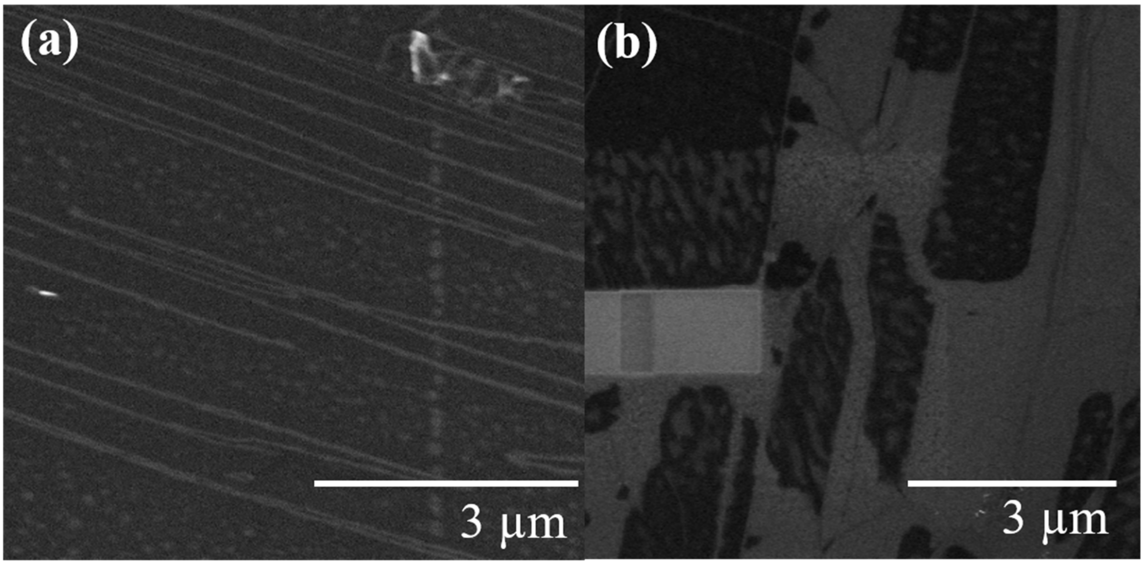

Figure SI3.1 Micrographs of Micron sized areas of a) Fresh surface; b) Aged surface showing the localized differences in secondary electron emissions (areas of bright contrast)

Figures SI3 show fresh and aged micrographs with typical emissions over a larger area of microns squared. Coverage values of specific grey values were obtained from micrographs for input into MC simulations.

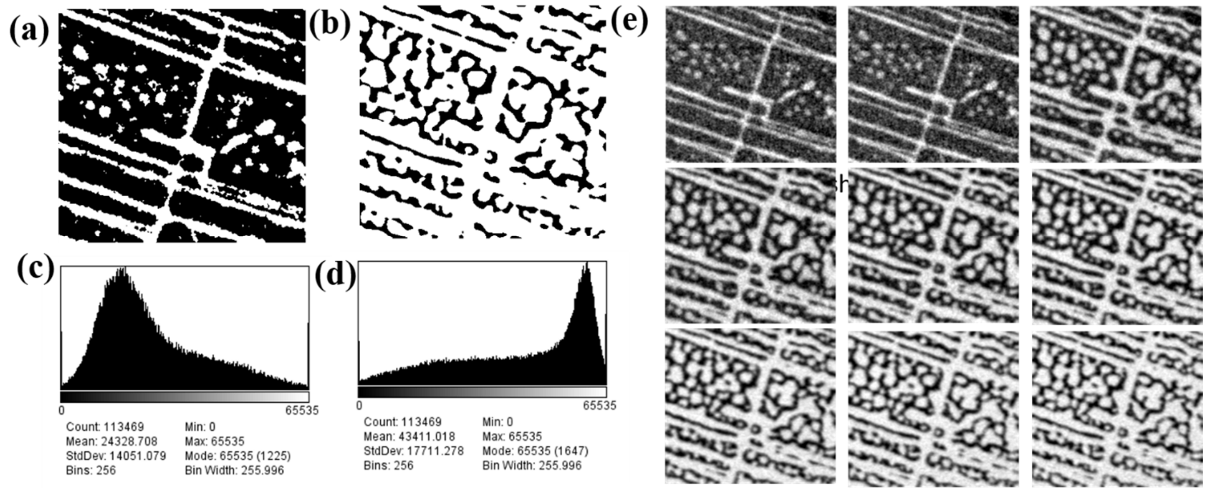

Figure SI3.2 Dynamic evolution of Primary contamination a) Binary image of Start (0.1 Cm-^{2} -lowest dose) of time series from Fresh HOPG Surface; b) Binary image of end (0.5 Cm-^{2} highest dose) of time series from a Fresh HOPG Surface; c) Histogram of Start image in a); d) Histogram of End image in b); e) Time series of micrographs <6eV illustrating Primary contamination.

Figure SI3.2 shows the binary images used to calculate the percentage grey level contributions from black to white. Figure SI3.2a) reveals the white contrast is 29.4 % area coverage and the end image. Figure SI3.2b) has a 73.9% area coverage of the white contrast. Figure SI3.2c) and d) are the associated histograms of a) and b). The end time series histogram shows emission predominantly in the “white” grey levels. This energy range is the sp^{3}-like Diamond. In the case of Diamond, the IMFP is extremely large, compared to the other components (HOPG and a-CH) as in Table SI3.4, leading to a larger signal in SE spectra due to the larger 'information volume".

Material	Energy (eV)	Inelastic Mean Free Path (A)
**HOPG Parallel**	10	27.6
**HOPG Perpendicular**	10	36.7
**a-CH**	10	43.0
**Diamond**	10	1144

Table SI3.4 Table of Inelastic mean free paths of Carbon allotropes

As shown in Figure 3 a) in the main document, there is contamination beneath the surface. Upon interaction with the primary electron beam, this will contribute to the sp^3 peak in the secondary electron emission range more than to the sp^2 peak and a-CH peak and so quite deep subsurface sp^3 areas will be visible in the SE image. Substantial dopant contrast has previously been demonstrated by energy selective SEM[14] . Our current work suggests that SE imaging using the P3 energy range is extremely responsive to sp^3 which is in contrast to Raman that show little sensitivity to sp^3. Thus SEHI is a complementary technique in the analysis of carbon materials. The drawback is of course that we have limited resolution when imaging with the sp^3 energy window unless the resolution is limited by the interaction volume, when very low primary electron beam energies are used.

SI 4 Raman Analysis

Raman Spectroscopy is a standard characterization method of carbon-based materials [15,16] This analytical technique depends on the inelastic scattering of light in a material of specific bond

vibrations. Raman spectra of carbon materials are mainly dominated by two features, the G and the D bands. The G band is related to the bond stretching of all pairs of sp^2 atoms in both rings and chains while the D band is related to the breathing modes of sp^2 atoms in the ring.[17] The intensity ratio between the G and the D bands (IG/ID) is generally used to define the degree of disorder. Figure SI4.1a) shows Raman spectra of 3 different HOPG surfaces- Fresh, Aged and EBID layer. With the exception of different intensities, the larger range of 0-4000 cm^{-1} reveals little difference between the three surfaces. Dou *et al*[18] have reported the visible Raman spectra of carbon-based materials spectra are due to sp^2 vibrations even in amorphous carbon structures which are dominated by sp^3 contributions. Dou further states that the IG/ID ratio appears to be not the best parameter to evaluate the amorphous nature of the investigated carbons, as it is only indirectly relates to the fraction of sp^3 sites. Figure SI.1b) shows the range of 1100- 1800 cm-1 which is where G and D are expected to be and small differences are shown. To understand these differences more, peak fitting according to Dou *et al* was completed as shown in Figure SI4.2.

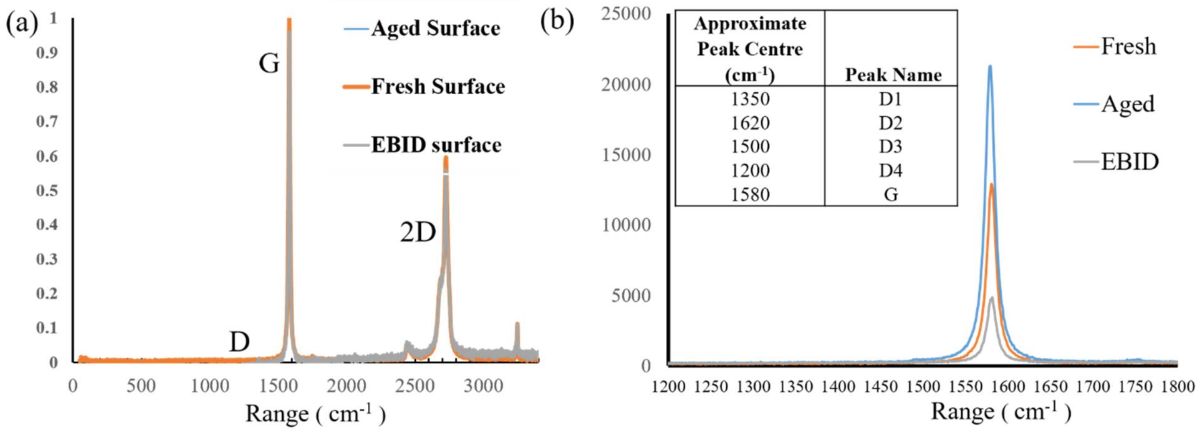

Figure SI 4.1 Raman Spectroscopy of fresh, aged and EBID surfaces of HOPG a) Range 2000-2400 cm^{-1}; b) Range of 1300-1800 cm^{-1} .

(a)

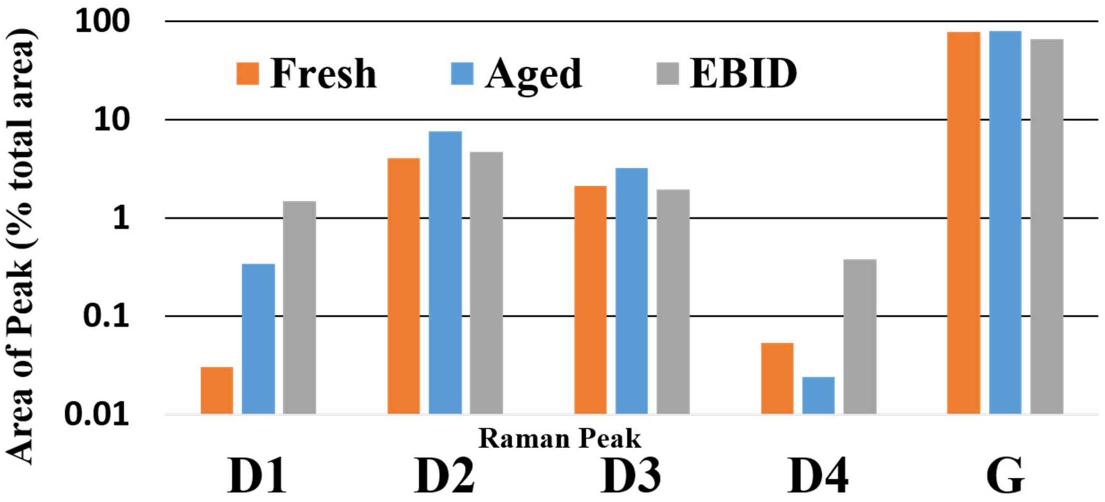

(b)

Peak Label	Peak Centre	Fresh	Aged	EBID
D1	1350	0.03	0.34	1.5
D2	1620	4.06	7.57	4.69
D3	1500	2.12	3.23	1.94
D4	1200	0.05	0.02	0.39
G	1580	77.5	79.02	65.75
	**Total D**	6.27	11.17	8.51
	**Id/Ig**	0.08	0.14	0.13

Figure SI 4.2 a) Peak fitting of Raman Peaks in 1300 to 1800 cm^{-1} of fresh, aged and EBID surface of HOPG; b) Table of Peak positions and the corresponding area of each peak.

Following Dou *et al* suggested deconvolution of the D and G bands into 5 components G, D1, D2, D3 and D4, the peaks of Figure SI 4.1b) were fitted. Figure SI4.2a) and b) shows the peak fitting of these 5 components. In the case of the G band, the 3 HOPG surfaces do not differ

whereas large differences are observed in D1 and D4. D1 is microcrystallite graphite and D4 is related to the CH termination groups. But this Raman analysis is limited by the lack of sp^3 signal.

SI 5 XPS Analysis

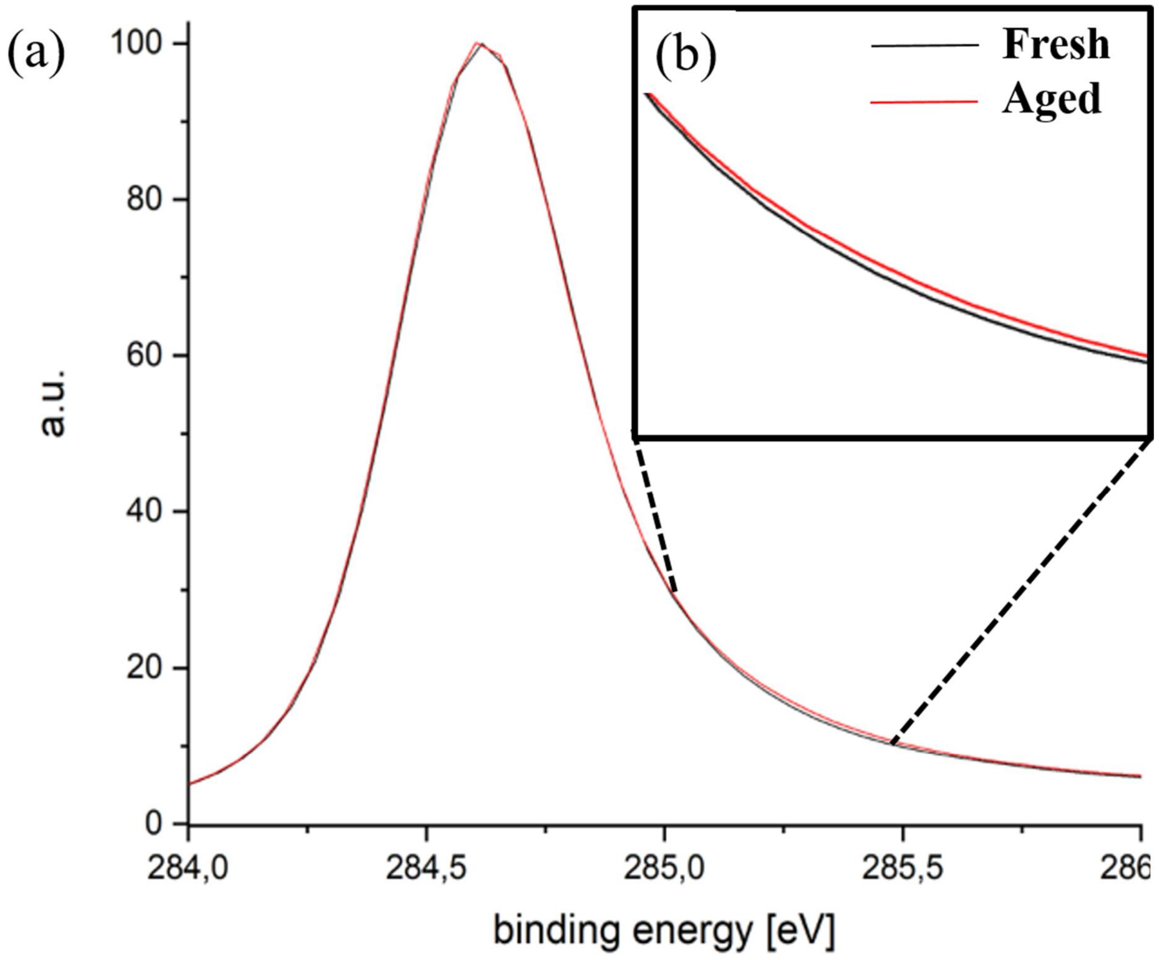

Figure SI5.1 a) XPS of Fresh and Aged HOPG surfaces; b) Inset is an enlargement of the asymmetric tail.

The XPS of the Fresh and Aged Samples resulted in HOPG_1 (fresh): 99.9 at% C, 0.1 at% O and HOPG_0 (aged): 99.4 at% C, 0.5 at% O, 0.1 at% Si. The C1s peaks of both samples are very

similar. C1s-Peak for both contains ~93 % sp^2 hybridized C and ≤ 1% sp^3 hybridized C and ~6% shake up. The D-parameter used was ~22.5 eV for freshly prepared HOPG and ~20 eV for aged HOPG [19]. Evaluation of C sp^2/ sp^3 content from the C 1s photoelectron spectrum requires resolving it into components representing different carbon chemical forms (C sp^2, C sp^3, other oxygen functional groups like hydroxyl C-OH, carbonyl C=O, carboxyl C-OOH) of characteristic binding energy (BE), full width at half maximum (FWHM) and BE separation values between C sp^2 and C sp^3 BE hybridisations. The ideal graphite exhibits C sp^2 BE at 284.5 eV.[19] The fitting of C 1s spectra should account for a known composition and structure of materials. Literature reports that values of BE, and C sp^2/sp^3 BE separation for C 1s spectra depend their structural disorder, defects, oxygen and hydrogen content -284.25–285.0 eV and _0.5–1.1 eV, respectively. A potential for ambiguous assignments of sp^3 C of C1s XPS spectra for carbon materials originates from the charging effect, which is caused by the disconnection of the pathway of electrons between sp^2C and sp^3C. [20] Care also is required as the different D parameter for different materials incur variations in the expected values.[19] In this work, the D parameter of 22% indicates sp^3 content <1%. Which is far below what the MC model suggest (in the main document). This could be due to the differences in IMFP of the photo electrons [21] (and see SI3) . In addition, the D parameter can vary an reach values as low as 18 after air exposure and it is also effected by grain sizes and orientation[22]. Thus, there is uncertainty in the actual sp^3 content present at the surface when quantification based on D-parameter is used. For instance, the sp^2/(sp^2+ sp^3) ratio for HOPG using surface sensitive Auger spectroscopy and D-parameter analysis resulted in only 89% sp^2 bonding but 96% when XPS data collected from the same sample where analyzed using the D parameter [20].

SI 6 EDX of PdAg films

Energy dispersive X-ray (EDX) spectroscopy is the currently the main analytical tool inside of a scanning electron microscope for elemental mapping. [23] In the case of beam-sensitive materials, this technique is limited because of its spatial resolution, ~ 1 μm. Our SE analysis is typically done at lower electron beam energy (≤1 kV) at this beam energy there is insufficient energy to excite characteristic X-rays. Here we performed EDX at 5kV to see what signals would be revealed. Please note that there is no coating on surface, which is typical in SEM analysis to enable probing the native surface but also limit the beam energy that can be applied.

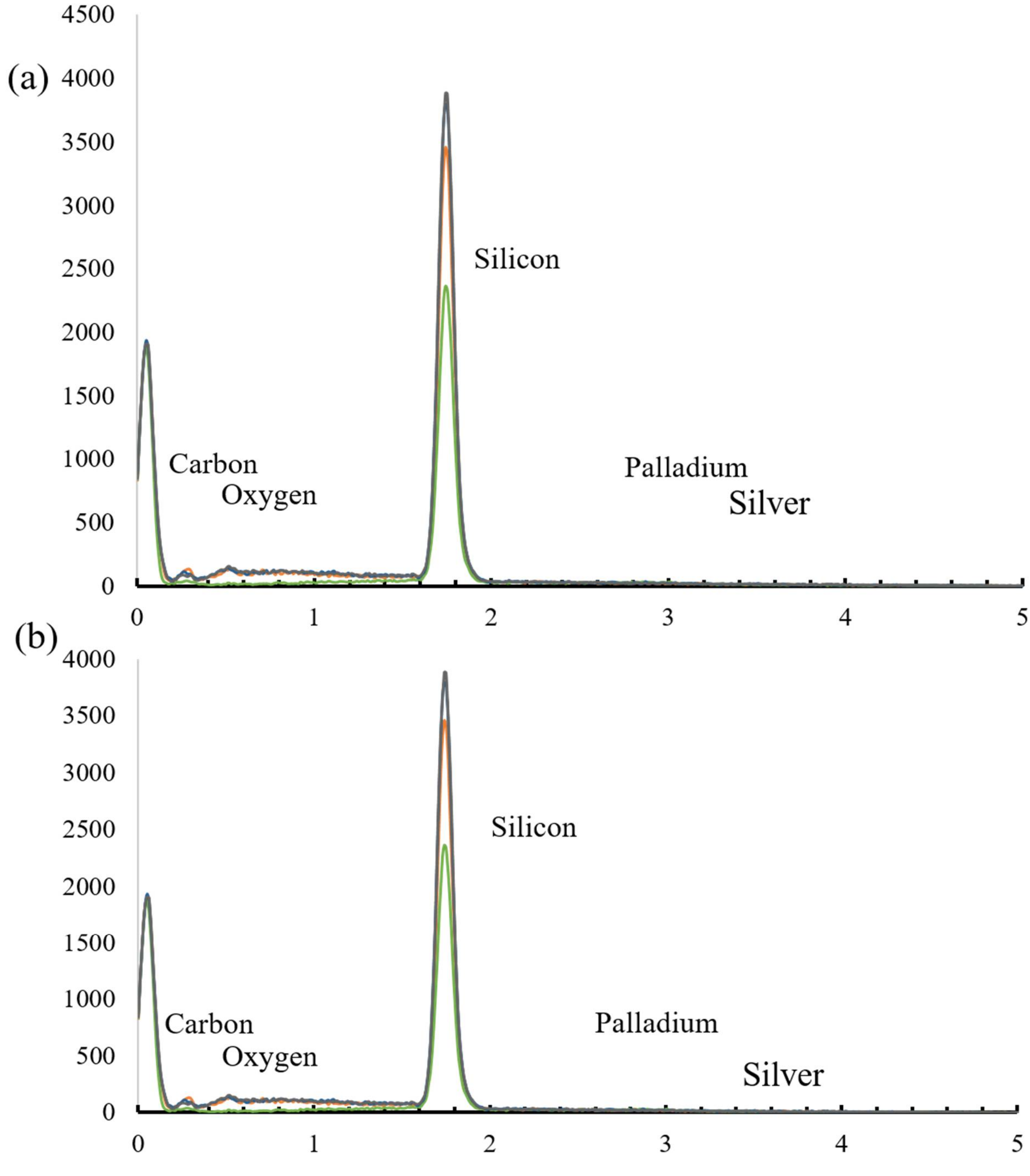

Figure SI 6.1 a) EDX Spectra of smooth regions of PdAg ROM film; b) EDX Spectra of PdAg ROM film of porous regions.

Spectrum	Morphology	Atomic %	Atomic %	Atomic %	Atomic %

Label		Pd	Ag	C	O
**5**	**Smooth**	27	9	0	64
**17**	**Smooth**	22	20	45	13
**26**	**Smooth**	4	17	0	79
**13**	**Substrate**	7	24	0	68
**15**	**Substrate**	0.2	0	0	99.8
**3**	**Porous**	59	16	18	7
**16**	**Porous**	58	18	18	6
**29**	**Porous**	51	27	17	6

Table SI 6.2 EDX calculations of atomic percentages of Pd, Ag, C and O in smooth and porous regions of PdAg ROM film and silicon substrate

EDX was performed on the Thick films to compare the elemental makeup of the porous and the smooth regions. Figure SI6.1a). The smooth regions have very similar EDX patterns and are dominated by the substrate (silicon) peak with very little Pd or Ag signal. But the SEHI imaging shows features on the surface which confirms the presence of a deposited film on the silicon substrate. Figure SI6.1b) shows the EDX spectra collected from porous regions. They all exhibit significant intensity in the Pd and Ag characteristic peaks energy region and indeed if porosity is high with a large topography then the silicon signal reduces to almost 0. With all EDX spectra, as the Pd increases so does the Carbon signal. Table SI6.2 show atomic percentages of the EDX spectra outputted after exclusion of Si – as such these are not true stoichiometries but show

relations between the Pd, Ag the C and O. The smooth regions seem to have a varied elemental make up with some Ag-rich regions and some Pd-rich regions and significant oxygen in all plots. With silicon excluded, the substrate EDX were predominantly Oxygen with the odd metal rich region which was most likely to be some spray contaminants from the print process. The porous regions shared trends with Pd being the more dominant than Ag and with each plot the carbon value was higher too. Leading us to conclude that EDX also reveals a relationship between the Pd and the Carbon within the film but only for sufficiently thick films.

References

[1] F. Mika, S. Konvalina, Krátký, I. Müllerová, *Trends* **2016**.

[2] T. Levesque, *A White Paper from XEI "Application of Plasma Cleaning Technology in Microscopy,"*

[3] N. Stehling, K. J. Abrams, C. Rodenburg, https://doi.org/10.15131/shef.data.7845044.v1.

[4] M. Dapor, A. Miotello, *Nucl. Instruments Methods Phys. Res. Sect. B Beam Interact. with Mater. Atoms* **2007**, *255*, 92.

[5] Q. Wan, K. J. Abrams, R. C. Masters, A. C. S. S. Talari, I. U. Rehman, F. Claeyssens, C. Holland, C. Rodenburg, *Adv. Mater.* **2017**, *29*, 1.

[6] N. Stehling, R. Masters, Y. Zhou, R. O'Connell, C. Holland, H. Zhang, C. Rodenburg, *MRS Commun*. **2018**, *8*, 226.

[7] N. Ueno, K. Seki, K. Sugita, H. Inokuchi, *Phys. Rev. B* **1991**, *43*, 2384.

[8] R. F. Willis, B. Fitton, *J. Vac. Sci. Technol.* **1972**, *9*, 651.

[9] J. Ferron, R. A. Vidal, N. Bajales, L. Cristina, R. A. Baragiola, *Surf. Sci.* **2014**, *622*, 83.

[10] A. Hoffman, S. Prawer, R. Kalish, *Phys. Rev. B* **1992**, *45*, 12736.

[11] V. A. Novolodski, O. M. Artamonov, S. A. Komolov, *Tech. Phys.* **1999**, *44*, 6.

[12] A. Otto, A Reihl, *Phys. Rev. B* **1990**, *41*, 9752.

[13] B. Lang, S. Tatarenko, *Solid State Commun.* **1979**, *31*, 303.

[14] C. Rodenburg, M. A. E. Jepson, E. G. T. Bosch, M. Dapor, *Ultramicroscopy* **2010**, *110*, 1185.

[15] M. A. Pimenta, G. Dresselhaus, M. S. Dresselhaus, L. G. Cançado, A. Jorio, R. Saito, *Phys. Chem. Chem. Phys.* **2007**, *9*, 1276.

[16] A. C. Ferrari, M. Katsnelson, L. Vandersypen, A. Loiseau, V. Morandi, A. Tredicucci, G. M. Williams, H. Hong, *Nanoscale* **2015**, *7*, 4598.

[17] L. Bokobza, J.-L. Bruneel, M. Couzi, *C* **2015**, *1*, 77.

[18] X. Dou, I. Hasa, D. Saurel, C. Vaalma, L. Wu, D. Buchholz, D. Bresser, S. Komaba, S. Passerini, *Mater. Today* **2019**, DOI 10.1016/j.mattod.2018.12.040.

[19] B. Lesiak, L. Kövér, J. Tóth, J. Zemek, P. Jiricek, A. Kromka, N. Rangam, *Appl. Surf. Sci.* **2018**, *452*, 223.

[20] A. Fujimoto, Y. Yamada, M. Koinuma, S. Sato, *Anal. Chem.* **2016**, *88*, 6110.

[21] B. Lesiak, L. Kövér, J. Tóth, J. Zemek, P. Jiricek, A. Kromka, N. Rangam, *Appl. Surf. Sci.* **2018**, *452*, 223.

[22] B. Lesiak, J. Zemek, J. Houdkova, A. Kromka, A. Józwik, *Anal. Sci.* **2010**, *26*, 217.

[23] L. Cinà, A. Di Carlo, F. Matteocci, C. Ducati, S. Cacovich, G. Divitini, *Nat. Energy* **2016**, *1*, 15012.
